# Fetal exposure to phthalates and bisphenols and DNA methylation at birth: the Generation R Study

**DOI:** 10.1186/s13148-022-01345-0

**Published:** 2022-10-10

**Authors:** Chalana M. Sol, Abigail Gaylord, Susana Santos, Vincent W. V. Jaddoe, Janine F. Felix, Leonardo Trasande

**Affiliations:** 1 The Generation R Study Group, Erasmus MC, University Medical Center Rotterdam, Rotterdam, The Netherlands; 2 Department of Pediatrics, Erasmus MC – Sophia Children’s Hospital, University Medical Center Rotterdam, Rotterdam, The Netherlands; 3 Department of Population Health, New York University School of Medicine, 403 East 34th Street, Room 115, New York City, NY 10016 USA; 4 Department of Pediatrics, New York University School of Medicine, 403 East 34th Street, Room 115, New York City, NY 10016 USA; 5 Department of Environmental Medicine, New York University School of Medicine, 403 East 34th Street, Room 115, New York City, NY 10016 USA; 6 New York Wagner School of Public Service, New York City, NY 10016 USA; 7 New York University Global Institute of Public Health, New York City, NY 10016 USA

**Keywords:** Bisphenols, Phthalates, Prenatal, Mixture, DNA methylation

## Abstract

**Background:**

Phthalates and bisphenols are non-persistent endocrine disrupting chemicals that are ubiquitously present in our environment and may have long-lasting health effects following fetal exposure. A potential mechanism underlying these exposure–outcome relationships is differential DNA methylation. Our objective was to examine the associations of maternal phthalate and bisphenol concentrations during pregnancy with DNA methylation in cord blood using a chemical mixtures approach.

**Methods:**

This study was embedded in a prospective birth cohort study in the Netherlands and included 306 participants. We measured urine phthalates and bisphenols concentrations in the first, second and third trimester. Cord blood DNA methylation in their children was processed using the Illumina Infinium HumanMethylation450 BeadChip using an epigenome-wide association approach. Using quantile g-computation, we examined the association of increasing all mixture components by one quartile with cord blood DNA methylation.

**Results:**

We did not find evidence for statistically significant associations of a maternal mixture of phthalates and bisphenols during any of the trimesters of pregnancy with DNA methylation in cord blood (all *p *values > 4.01 * 10^–8^). However, we identified one suggestive association (*p *value < 1.0 * 10^–6^) of the first trimester maternal mixture of phthalates and bisphenols and three suggestive associations of the second trimester maternal mixture of phthalates and bisphenols with DNA methylation in cord blood.

**Conclusions:**

Although we did not identify genome-wide significant results, we identified some suggestive associations of exposure to a maternal mixture of phthalates and bisphenols in the first and second trimester with DNA methylation in cord blood that need further exploration in larger study samples.

**Supplementary Information:**

The online version contains supplementary material available at 10.1186/s13148-022-01345-0.

## Background

Endocrine disrupting chemicals (EDCs) such as phthalates and bisphenols are used in many consumer products such as cosmetics and plastic food packaging [1, 2]. As a result, throughout their lives, humans are continuously exposed to a mixture of these endocrine disruptors. Previous studies have shown associations of exposure to endocrine disruptors with cardiometabolic health, insulin resistance and fertility in humans [3–6]. As phthalates and bisphenols are able to cross the placenta, exposure begins in utero [7, 8]. Fetal life has been suggested to be a sensitive period for exposure to environmental factors that determine life-long patterns of health and disease, as suggested in the Developmental Origins of Health and Disease framework [9]. Previous studies of EDC exposure during fetal life with childhood health have also shown an association of these exposures with birth weight, neurodevelopment and cardiometabolic risk factors during childhood, among others [3, 10–13].

One of the proposed mechanisms by which phthalates and bisphenols could affect health is by influencing DNA methylation [14]. Results from studies that have examined the association between phthalate or bisphenol exposure during fetal life and DNA methylation in humans are not consistent. Varying associations of fetal exposure to phthalates or bisphenol A were found with DNA methylation of the insulin-like growth factor 2 (*IGF2*) gene in candidate-gene studies [15–19]. These studies assessed DNA methylation in different tissues. In the study by Montrose et al., DNA methylation was assessed in cord blood, while in two other studies, placental tissue was used [16, 17, 19]. Finally, in two other studies, whole blood during childhood was used [15, 18]. As DNA methylation is tissue- and age-specific, results of these studies are not directly comparable. Several other studies of prenatal bisphenol and phthalate exposure, using an epigenome-wide approach, have identified differential DNA methylation at multiple CpG sites in cord blood in relation to exposure to different phthalates or bisphenols [20–25]. In addition, one study explored the associations of prenatal exposure to phthalates with DNA methylation in peripheral blood and buccal epithelial cells during childhood [26]. When exploring the associations with DNA methylation in cord blood, only the studies by Miura et al. and Petroff et al. included more than 70 mother–child pairs and the studies by Chen et al. and Petroff et al. were the only studies that included a phthalate [20, 22, 24, 25]. Most previous studies that assessed the associations of maternal phthalate or bisphenol urine concentrations with DNA methylation focused on one or a few phthalates or bisphenols and did not consider joint effects of the exposures [15–30]. This is an oversimplification, since humans are exposed to a mixture of different chemicals that could influence each other and thus could have synergistic or antagonistic effects. Focusing on only one single exposure might not fully elucidate the total mixture-effect of endocrine disruptors during pregnancy.

To overcome this limitation, we investigated the associations of fetal exposure to a mixture of phthalates and bisphenols, using a novel statistical approach, with DNA methylation in offspring [31]. We measured phthalate and bisphenol concentrations at three time points during pregnancy in spot urine samples obtained from 306 participants in a Dutch population-based cohort study and measured DNA methylation in an epigenome-wide association study (EWAS) in cord blood in their children.

## Methods

### Design

This study was embedded in the Generation R Study, a population-based prospective cohort study starting during early fetal life in Rotterdam, the Netherlands [32]. The study has been approved by the Medical Ethical Committee of the Erasmus MC, University Medical Center Rotterdam. Written informed consent was obtained for all participants.

Mother–child pairs were included in this exploratory study if bisphenols and phthalates urine concentrations were available at all three time points during pregnancy, which was in a subsample of 1379 mothers of the full Generation R Study, and if DNA methylation was measured in cord blood collected at birth. DNA methylation data were collected in a subsample of children participating in the full Generation R Study, consisting of participants with parents born in the Netherlands. A total of 306 mothers and their children were included in the current analyses (Additional file 1: Fig. S1).

### Phthalate and bisphenol measurements

Between February 2004 and July 2005, women were invited to our research facility during early (median 12.6 weeks, interquartile range (IQR) 2.0 weeks), mid (median 20.4 weeks, IQR 1.0 weeks) and late pregnancy (median 30.2 weeks, IQR 1.3 weeks), at which time they provided a spot urine sample. The analyses of the phthalate, bisphenol and creatinine concentrations were performed at the Wadsworth Center, New York State Department of Health, Albany, New York, USA. Collection, transportation and analysis of these urine samples have been previously described [33]. Of all measured phthalates and bisphenols, we included those in the assessment that had less than 25% of their concentrations during all trimesters below the limit of detection (LOD). Phthalates and bisphenols with more than 25% of the samples below the limit of detection were thus not included in the further analysis. Concentrations below the LOD were substituted by LOD divided by the square root of 2 (LOD/√2) [34]. To account for urinary dilution in the analysis, all urine concentrations of phthalates and bisphenols were converted to µmol/g creatinine. An overview of the concentrations of included phthalates and bisphenols is presented in Table 1. (Non-participants are shown in Additional file 1: Table S1.) Finally, to reduce skewedness of the distributions, the phthalate and bisphenol urine concentrations were natural log transformed. As a sensitivity analysis, all analyses were repeated using the averaged concentrations over pregnancy.

**Table 1:** Urine concentrations of phthalates and bisphenols, specified per trimester

	LOD (nmol/L)	First trimester		Second trimester		Third trimester	
		Median (25–75th percentile)	Percentage < LOD	Median (25–75th percentile)	Percentage < LOD	Median (25–75th percentile)	Percentage < LOD
Phthalic acid (PA) (nmol/L)	6.68	349.2 (195.4–844.3)	0.3	953.2 (381.6–1558.6)	0	345.7 (187.1–715.6)	0.3
Monomethylphthalate (mMP) (nmol/L)	0.33	28.3 (14.8–52.4)	0.3	18.5 (9.2–34.1)	0.3	16.8 (9.4–36.6)	1.0
Monoethylphthalate (mEP) (nmol/L)	0.31	671.5 (198.0–2412.9)	0	330.5 (123.2–1057.6)	0	591.3 (207.8–1775.7)	0
Mono-isobutylphthalate (mIBP) (nmol/L)	0.40	84.2 (38.3–157.8)	0	35.6 (18.9–66.4)	0	58.9 (33.6–115.0)	0.3
Mono-*n*-butylphthalate (mBP) (nmol/L)	0.63	68.5 (30.8–124.5)	0.3	41.0 (24.4–75.1)	0	45.7 (23.9–78.8)	0
Monobenzylphthalate (mBzBP) (nmol/L)	0.59	24.6 (8.8–43.6)	6.9	17.4 (7.2–33.6)	2.9	9.9 (3.3–19.1)	3.6
Mono-(2-ethyl-5-carboxy-pentyl)phthalate (mECPP) (nmol/L)	0.94	46.8 (26.3–92.9)	0	33.2 (18.7–59.2)	0	51.7 (26.8–90.3)	0
Mono-(2-ethyl-5-hydroxy-hexyl)phthalate (mEHHP) (nmol/L)	0.27	35.1 (16.5–73.8)	0	19.1 (10.6–36.8)	0	33.0 (15.9–58.0)	0
Mono-(2-ethyl-5oxohexyl)phthalate (mEOHP) (nmol/L)	0.14	22.6 (10.4–45.7)	0	26.5 (14.2–56.7)	0	22.5 (12.1–43.4)	0
Mono-[(2-carboxymethyl)-hexyl]phthalate (mCMHP) (nmol/L)	0.13	42.4 (23.3–74.3)	0	12.3 (7.1–23.4)	0.3	9.1 (5.0–17.4)	0
Mono(3-carboxypropyl)phthalate (mCPP) (nmol/L)	0.03	5.2 (3.0–10.4)	0	3.6 (2.0–6.6)	0	6.6 (3.7–12.1)	0
Bisphenol A (BPA) (nmol/L)	0.66	6.2 (1.6–14.8)	18.0	5.2 (2.4–12.2)	8.2	6.0 (2.9–11.0)	9.2

Values represent medians (25–75th percentiles). Absolute urine concentration of the limit of detection (in nmol/L urine) and individual exposures (in nmol/L urine) with concentrations below the limit of detection imputed as limit of detection/square root of 2. Only phthalates and bisphenols that have at least 75% detection in all trimesters are presented in this table. Individual exposures assessed but not included in the analysis in this study due to less than 75% of concentrations above the limit of detection in all trimesters include monoisononylphthalate, monocyclohexylphthalate, monooctylphthalate, mono-(8-methyl-1-nonyl)phthalate, mono-hexylphthalate, mono-2-heptylphthalate, mono-(7-carboxy-*n*-heptyl)phthalate, bisphenol S, bisphenol Z, bisphenol B, bisphenol F, bisphenol AP, bisphenol AF and bisphenol P

*LOD* limit of detection

### DNA methylation measurement

After birth, cord blood was drawn by the attending physician or midwife. From these samples, DNA was extracted using the salting-out method. After bisulfite conversion of 500 ng DNA using the EZ-96 DNA Methylation kit (Shallow) (Zymo Research Corporation, Irvine, USA), samples were processed with the Illumina Infinium HumanMethylation450 BeadChip (Illumina Inc., San Diego, USA). At the time of processing the cord blood samples from the Generation R Study, the MethylationEPIC BeadChip was not available yet and whole-genome bisulfite sequencing was not feasible for high-throughput analysis in population studies. Beta values, which represent the ratio of methylated signal relative to the total (methylated and unmethylated) signal per CpG, were calculated. Quality control and normalization were performed using the CPACOR workflow [35]. Probes with a detection *p *value ≥ 1.0 * 10^–16^ were set to missing. Intensity values were quantile normalized. Arrays with observed technical problems or with a mismatch between sex of the proband and sex determined by the intensities of the X- and Y-chromosome were removed from the analysis. Only arrays with a call rate > 95% per sample were processed further, and DNA methylation beta values outside the range of (25th percentile – 3 * IQR, 75th percentile + 3 * IQR) were set to missing. Probes on the X and Y chromosomes were excluded from the dataset. Additionally, we removed cross-reactive probes, leaving information on 415,786 CpGs at birth [36, 37]. Probes that map to DNA containing a single nucleotide polymorphism (SNP), repetitive sequence elements or DNA harboring an insertion or deletion were flagged, but not removed [36, 37].

### Covariates

Information on potential confounders was collected using questionnaires during pregnancy. Potential confounders were chosen based on their known association with both phthalate and bisphenol exposure and with DNA methylation. Included covariates were maternal age at inclusion, maternal pre-pregnancy body mass index (BMI), maternal educational level and maternal smoking habits (sustained versus non-sustained smoking during pregnancy). Child sex was obtained from midwife and hospital records. Sample plate number was included in the analysis to correct for batch effects. Plates with fewer than two participants were grouped together, which was done for six plates, as not all mother–child pairs from the Generation R Study in whom DNA methylation was measured in cord blood had information on the maternal phthalate and bisphenol urine concentrations during pregnancy available. White blood cell composition was estimated with the Salas method for cord blood, which included B-lymphocytes, CD4+ T-lymphocytes, CD8+ T-lymphocytes, granulocytes, monocytes, natural killer cells and nucleated red blood cells. Ethnicity and use of folic acid supplements were not assessed as potential confounders in this study, since all participants were of European ancestry and almost all participants (93.6%) used folic acid supplements.

### Statistical analysis

Missing data for covariates (ranging between 0.3 and 11.4%) were imputed ten times by the Multivariate Imputation by Chained Equations (MICE) method in R. Imputation was successful for all covariates, and the last imputed dataset was used for all analysis. When all association analyses were repeated with a random other dataset as a sensitivity analysis, there were no differences in the reported associations. To assess the joint effects of the phthalate and bisphenol mixture in a specific trimester, we used the quantile-based g-computation approach from the *qgcomp* package in R [31]. In quantile g-computation, the exposures of interest are quantized (e.g., transformed into categories of exposure), after which the effect of increasing all exposures by one quantile simultaneously is evaluated by estimating the parameters of a marginal structural model given the joint intervention on the exposures. The main advantages of this method are the easy interpretation of the association and the absence of a need for directional homogeneity. Using this method, we were able to estimate the joint effect of increasing all mixture components by one quartile.

To examine associations of the chemical mixture with DNA methylation in cord blood, we first ran basic linear models adjusting for child sex, estimated cell types and batch. We then ran fully adjusted linear models adjusting for child sex, maternal education, maternal smoking during pregnancy, maternal age at inclusion, maternal pre-pregnancy BMI, estimated cell types and batch.

We used Bonferroni correction (*p *value cutoff < 4.01 * 10^–8^ based on an original *p *value cutoff of 0.05 and 415,786 tests per trimester, giving a total of 1,247,358 tests for the three trimesters) as the primary cutoff to assess statistical significance. Additionally, we defined suggestive associations based on a *p *value cutoff of < 1.0 * 10^–6^, as we feared to be too rigorous in dismissing potential associations that did not reach statistical significance due to the exploratory nature of this study. To provide a more comprehensive overview of the results, we present all associations with a *p *value cutoff of < 1.0 * 10^–5^ in the supplemental tables. We performed a priori defined exploratory analyses stratified on sex, as it has been hypothesized that exposure to endocrine disruptors could have different effects based on sex [38].

## Results

### Subject characteristics

Compared to non-participants, participating mothers in the present study were more often of European ancestry, highly educated and were less likely to sustain smoking during pregnancy (Table 2). Almost all participants used folic acid supplementation during early pregnancy. Most phthalate concentrations were higher among non-participants than among participants, but bisphenol A concentrations during first trimester were lower among non-participants (Table 1 and Additional file 1: Table S1).

**Table 2:** Participant and non-participant characteristics

	Participants (three trimesters)	Non-participants
	*n* = 306	*n* = 1 073
Maternal characteristics		
Age at enrollment, mean (SD) (years)	32.1 (3.9)*	30.1 (5.0)*
Ethnicity, *n* (%)		
European ancestry	298 (97.4%)*	455 (42.9%)*
Non-European ancestry	8 (2.6%)*	605 (57.1%)*
Education, *n* (%)		
Low-middle	92 (30.2%)*	564 (55.6%)*
High	213 (69.8%)*	450 (44.4%)*
Pre-pregnancy BMI, median (95% range) (kg/m^2^)	22.6 (18.7–34.4)	22.7 (18.4–35.1)
Folic acid supplementation, *n* (%), yes	233 (93.6%)*	654 (76.9%)*
Smoking sustained during pregnancy, *n* (%), yes	24 (8.9%)*	155 (15.9%)*
Alcohol consumption sustained during pregnancy (any), *n* (%), yes	202 (55.8%)*	340 (35.1%)*
Child characteristics		
Gender (boys), *n* (%)	160 (52.3%)	536 (50.0%)
Birth weight, mean (SD) (g)	3556 (468)*	3425 (503)*
Gestational age at birth, mean (SD) (weeks)	40.3 (1.3)*	40.0 (1.5)*

Values represent numbers (valid percent), mean (SD) or median (95% range)

*SD* standard deviation

*p value < 0.05

### Associations of exposure to a mixture of endocrine disruptors and DNA methylation in cord blood

**Fig. 1:**
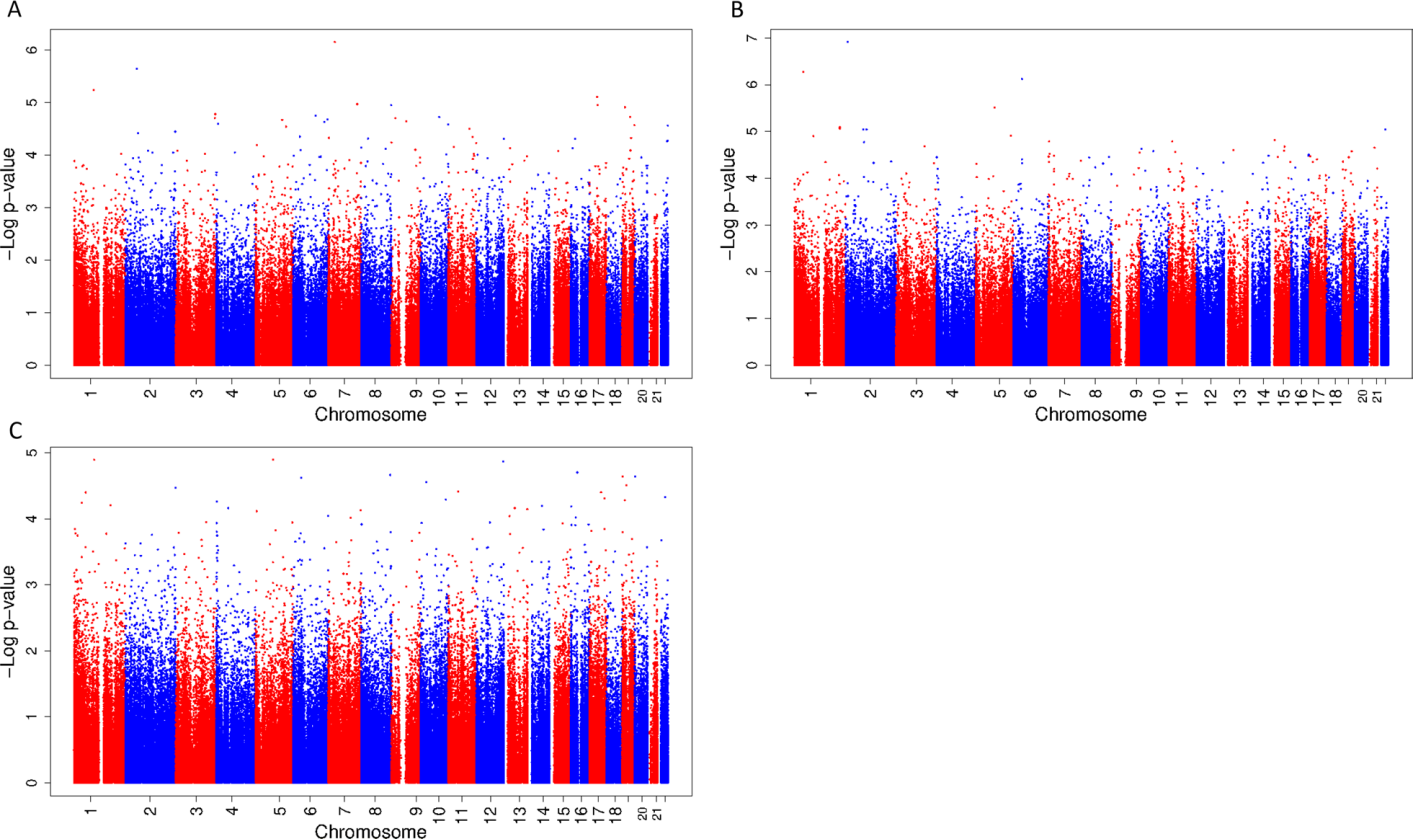
Manhattan plot of associations between a mixture of phthalates and bisphenols during first, second and third trimester with DNA methylation at birth. Manhattan plot of associations between a mixture of phthalates and bisphenols during first (**A**), second (**B**) and third (**C**) trimester with DNA methylation at birth in the total population. In all Manhattan plots, the *x*-axis represents the autosomal chromosomes, the *y*-axis represents the −log_10_ of the *p *value and the dots represent CpGs

In the total study population, there were no significant associations of fetal exposure to a mixture of phthalates and bisphenols during either first, second or third trimester with DNA methylation in cord blood. (Figure 1A–C shows the Manhattan plots, CpGs with a *p *value < 1.0 * 10^–6^ are presented in Table 3, and CpGs with a *p *value < 1.0 * 10^–5^ are presented in Additional file 1: Table S2.) There were a few suggestive associations. The strongest association in the first trimester was found with decreased DNA methylation of cg05058973 (effect − 1.20 * 10^–2^ (standard error (SE) 2.37 * 10^–3^) per quartile increase in the mixture, *p *value 7.08 * 10^–7^), which maps to the growth hormone-releasing hormone receptor *(GHRHR)* gene. In the second trimester, we found a suggestive association with an increase in DNA methylation of cg00141688, located near the hippocalcin-like 1 (*HPCAL1*) gene, and cg15961211, which is close to the family with sequence similarity 183 member A (*FAM183A*) gene (effect per quartile increase in the mixture: 1.59 * 10^–2^ (SE 2.93 * 10^–3^), *p *value 1.21 * 10^–7^ and 3.65 * 10^–3^ (SE 7.11 * 10^–4^), *p *value 5.32 * 10^–7^, respectively) and with a decrease in DNA methylation of cg20840540, which is close to transcriptional-regulating factor 1 (*TRERF1*) (effect per quartile increase in the mixture − 1.28 * 10^–2^ (SE 2.52 * 10^–3^), *p *value 7.54 * 10^–7^). The analysis for the third trimester mixture showed no suggestive associations. Results for the models unadjusted for demographic covariates were comparable to those from the fully adjusted model (Additional file 1: Table S3 and Fig. S2).

**Table 3:** CpGs with *p *values < 1.0 * 10^–6^ from epigenome-wide association study of a mixture of phthalates and bisphenols in maternal urine during first, second and third trimester and DNA methylation in cord blood

	CpG	Chr	Position	Gene	Effect	SE	*p *value	Flag^#^
First trimester	cg05058973	7	31002599	*GHRHR*	− 1.20 * 10^–2^	2.37 * 10^–3^	7.08 * 10^–7^	0
Second trimester	cg00141688	2	10517352	*HPCAL1*	1.59 * 10^–2^	2.93 * 10^–3^	1.21 * 10^–7^	0
	cg15961211	1	43613440	*FAM183A*	3.65 * 10^–3^	7.11 * 10^–4^	5.32 * 10^–7^	0
	cg20840540	6	42363749	*TRERF1*	− 1.28 * 10^–2^	2.52 * 10^–3^	7.54 * 10^–7^	0

There were no CpGs presented for third trimester, as none reached our uncorrected *p *value cutoff of < 1.0 * 10^–6^. There were no associations that reached significance (*p *value < 0.05) after further FDR-adjustment of the *p *value for multiple testing including the three trimesters

^#^We have indicated probes that map to DNA containing a single nucleotide polymorphism (SNP), repetitive sequence elements or DNA harboring an insertion or deletion with a ‘1’ in this column

**Fig. 2:**
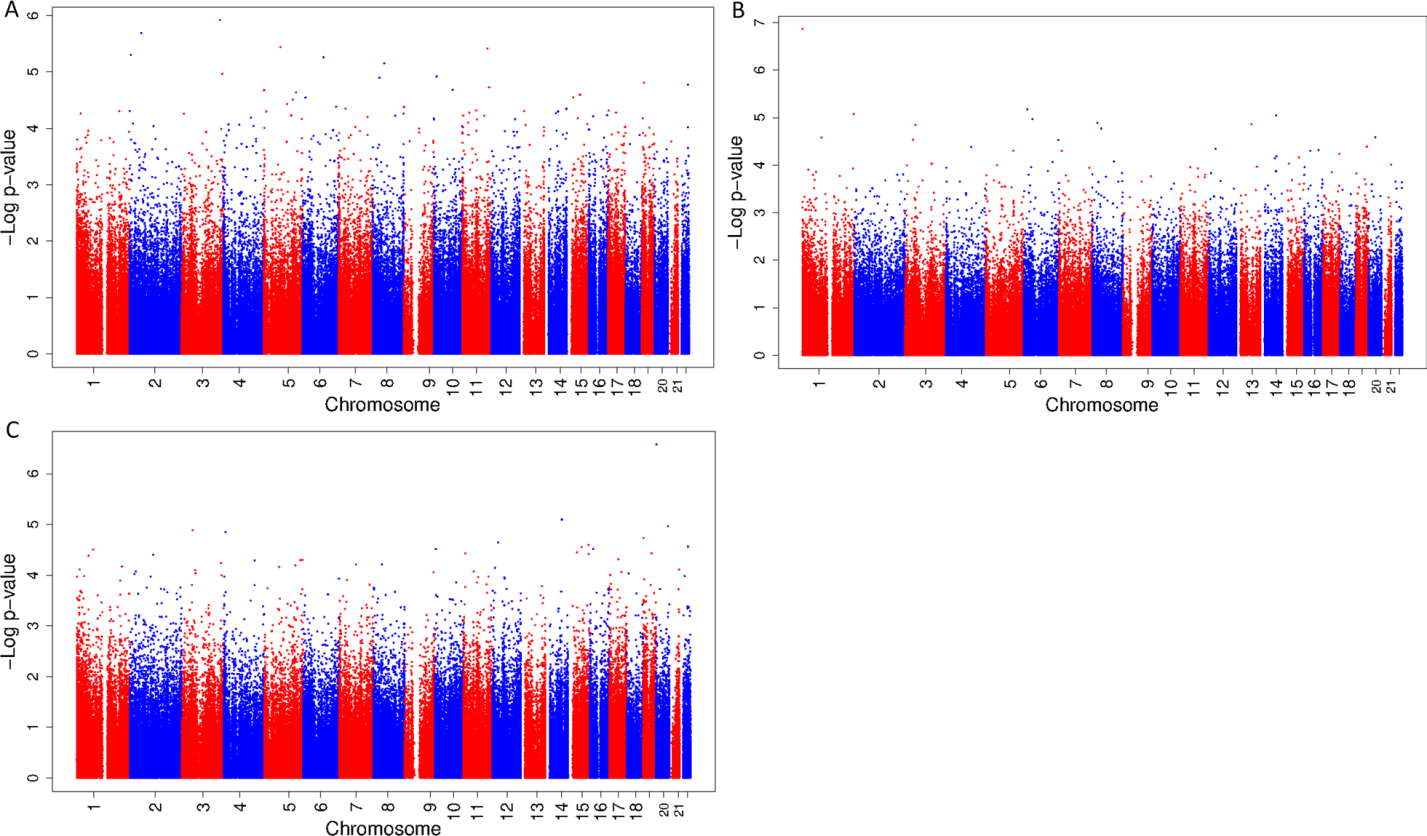
Manhattan plot of associations between a mixture of phthalates and bisphenols during first, second and third trimester with DNA methylation at birth among boys. Manhattan plot of associations between a mixture of phthalates and bisphenols during first (**A**), second (**B**) and third (**C**) trimester with DNA methylation at birth among boys. In all Manhattan plots, the *x*-axis represents the autosomal chromosomes, the *y*-axis represents the −log_10_ of the *p *value and the dots represent CpGs

**Fig. 3:**
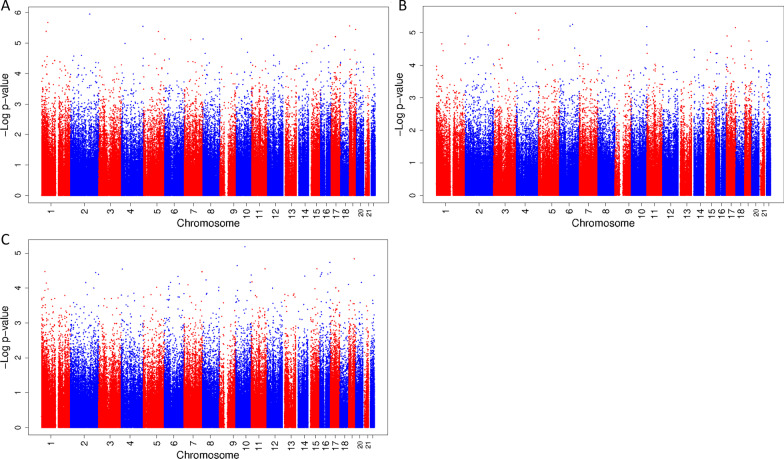
Manhattan plot of associations between a mixture of phthalates and bisphenols during first, second and third trimester with DNA methylation at birth among girls. Manhattan plot of associations between a mixture of phthalates and bisphenols during first (**A**), second (**B**) and third (**C**) trimester with DNA methylation at birth among girls. In all Manhattan plots, the *x*-axis represents the autosomal chromosomes, the *y*-axis represents the −log_10_ of the *p *value and the dots represent CpGs

In the explorative stratified analyses among boys, there were no statistically significant associations of exposure to a mixture of phthalates and bisphenols during pregnancy with cord blood DNA methylation at birth. (Figure 2A–C shows the Manhattan plots, CpGs with a *p *value < 1.0 * 10^–6^ are presented in Table 4, and CpGs with a *p *value < 1.0 * 10^–5^ are presented in Additional file 1: Table S4.) However, there were some suggestive associations of exposure to the mixture during second trimester with a decrease in DNA methylation of cg03764767, which is located in the peroxisomal biogenesis factor 10 (*PEX10*) gene, and exposure to the mixture during third trimester with an increase in DNA methylation of cg23462052, located close to the small nuclear ribonucleoprotein polypeptides B and B1 (*SNRPB*) gene. (Figure 2A–C shows the Manhattan plots, CpGs with a *p *value < 1.0 * 10^–6^ are presented in Table 4, and CpGs with a *p *value < 1.0 * 10^–5^ are presented in Additional file 1: Table S4.) Among girls, there were no statistically significant or suggestive associations of exposure to the mixture during any of the trimesters with cord blood DNA methylation. (Figure 3A–C shows the Manhattan plots, and CpGs with a *p *value < 1.0 * 10^–5^ are presented in Additional file 1: Table S5.)

**Table 4:** CpGs with *p *values < 1.0 * 10^–6^ from epigenome-wide association study of a mixture of phthalates and bisphenols in maternal urine during first, second and third trimester and DNA methylation in cord blood among boys

	CpG	Chr	Position	Gene	Effect	SE	*p *value	Flag^#^
Second trimester	cg03764767	1	2338210	*PEX10*	− 1.85 * 10^–2^	3.33 * 10^–3^	1.36 * 10^–7^	1
Third trimester	cg23462052	20	2452871	*SNRPB*	1.27 * 10^–2^	2.36 * 10^–3^	2.65 * 10^–7^	1

There were no CpGs presented for first trimester, as none reached our uncorrected *p *value cutoff of < 1.0 * 10^–6^. There were no associations that reached significance (*p *value < 0.05) after further FDR-adjustment of the *p *value for multiple testing including the three trimesters

^#^We have indicated probes that map to DNA containing a single nucleotide polymorphism (SNP), repetitive sequence elements or DNA harboring an insertion or deletion with a ‘1’ in this column

**Fig. 4:**
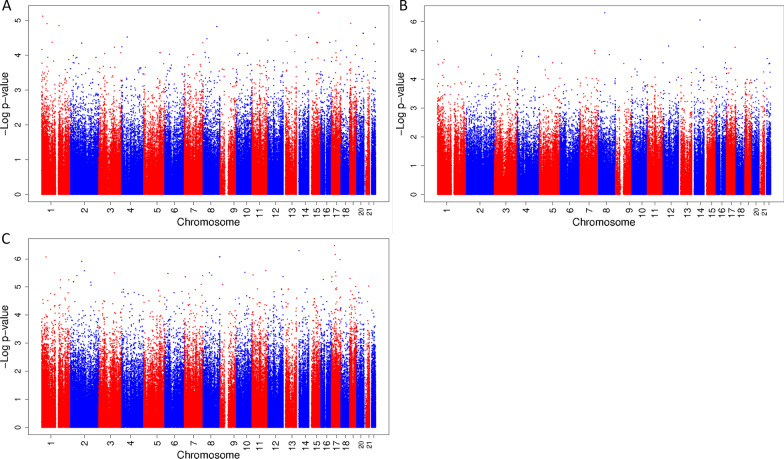
Manhattan plot of associations between a mixture of phthalates and bisphenols averaged over pregnancy with DNA methylation at birth in the total population and among boys and girls specifically. Manhattan plot of associations between a mixture of phthalates and bisphenols averaged over pregnancy in the total population (**A**), among boys (**B**) and among girls (**C**) with DNA methylation at birth. In all Manhattan plots, the *x*-axis represents the autosomal chromosomes, the *y*-axis represents the −log_10_ of the *p *value and the dots represent CpG

In the sensitivity analysis, in which we used the averaged mixture during pregnancy, there were no significant associations for fetal exposure to the mixture of phthalates and bisphenols in the total study population. (Figure 4A–C shows the Manhattan plots, CpGs with a *p *value < 1.0 * 10^–6^ are presented in Table 5, and CpGs with a *p *value < 1.0 * 10^–5^ are presented in Additional file 1: Table S6.) Among boys, there were two CpGs that had a suggestive association and among girls, five CpGs had a suggestive association. These associations did not overlap with the suggestive associations we identified in the trimester-specific analyses.

**Table 5:** CpGs with *p *values < 1.0 * 10^–6^ from epigenome-wide association study of a mixture of phthalates and bisphenols in maternal urine averaged over pregnancy and DNA methylation in cord blood among boys and girls specifically

	CpG	Chr	Position	Gene	Effect	SE	*p *value	Flag^#^
Boys	cg20400361	8	55,014,040	*LYPLA1*	9.50 * 10^–3^	1.80 * 10^–3^	4.93 * 10^–7^	0
	cg00025138	14	71,275,917	*MAP3K9*	− 2.57 * 10^–3^	5.01 * 10^–4^	8.64 * 10^–7^	0
Girls	cg13344757	17	26904381	*ALDOC*	1.20 * 10^–2^	2.24 * 10^–3^	3.40 * 10^–7^	0
	cg04913443	14	21566084	*ZNF219;C14orf176*	− 7.00 * 10^–3^	1.33 * 10^–3^	5.13 * 10^–7^	0
	cg11723896	17	34136427	*TAF15*	− 1.05 * 10^–2^	2.02 * 10^–3^	6.95 * 10^–7^	1
	cg10074813	8	144637872	*GSDMD*	1.51 * 10^–2^	2.93 * 10^–3^	8.43 * 10^–7^	1
	cg15504459	1	38478291	*UTP11L*	− 8.02 * 10^–3^	1.55 * 10^–3^	8.60 * 10^–7^	0

There were no CpGs presented for the total group, as none reached our uncorrected *p *value cutoff of < 1.0 * 10^–6^

^#^We have indicated probes that map to DNA containing a single nucleotide polymorphism (SNP), repetitive sequence elements or DNA harboring an insertion or deletion with a ‘1’ in this column

## Discussion

### Main findings

In this relatively small population-based cohort study including children of European ancestry, using a novel statistical method for the analysis of joint mixture effects of exposure to phthalates and bisphenols during each trimester of pregnancy, we found that exposure to a mixture of phthalates and bisphenols was not significantly associated with DNA methylation in cord blood. However, there were some suggestive associations for exposure in the first and second trimester.

### Comparison to the literature

In the total group, we found that exposure to the mixture of phthalates and bisphenols during the first trimester has a suggestive association with a decrease in DNA methylation of cg05058973, which is close to the *GHRHR* gene. This receptor is mainly expressed in the anterior pituitary gland, and in vitro studies have shown that it plays a role in the regulation of growth hormone, which influences protein and fat metabolism [39]. From animal studies, we know that expression of the *GHRHR* gene could be influenced by estrogen [40]. Exposure to the mixture during second trimester had a suggestive association with an increase in DNA methylation of cg00141688, which is located near *HPCAL1*. It has been reported that neonatal exposure to estrogens or BPA led to an increase in DNA methylation of the promoter of *HPCAL1* in the prostate gland of rats with persisting effects during life [41]. We additionally found a suggestive association of exposure to the mixture during second trimester with an increase in DNA methylation of cg15961211, which is located near the *FAM183A* gene. This gene has no known function in relation to exposure to phthalates or bisphenols. Exposure to the mixture during the second trimester also had a suggestive association with a decrease in DNA methylation of cg20840540, which is located close to *TRERF1*, a gene that interacts with the progesterone receptor after its activation by progesterone [42]. Among boys, second trimester exposure to the mixture showed a suggestive association with a decrease in DNA methylation of cg03764767, which is within the *PEX10* gene, which has been indicated as having a role in male fertility as it is important for spermatocyte development [43]. Also among boys, there was some indication that exposure to the mixture during third trimester could be associated with an increase in DNA methylation of cg23462052, which is near the *SNRPB* gene that has no known function related to our exposures.

The differentially methylated CpG sites identified in this study have not been associated with prenatal BPA and phthalate exposure in previous studies in humans, although a direct comparison of this study with previous literature is difficult, as none of the previous studies studied mixture effects using quantile g-imputation. Apart from implementing a mixtures model in this analysis, this study also differed from previous studies in other ways. First, several of the studies that have shown associations between fetal phthalate and bisphenol exposure and DNA methylation at birth have conducted targeted analyses using pyrosequencing [19, 44, 45]. Different methods have also been used to model chemical exposure, including exposure in the 25th percentile versus the 75th percentile, comparing subjects above and below the 75th percentile, or including the chemicals separately as continuous variables [17, 20–24, 27, 29, 44, 46]. To our knowledge, the only other study that used a chemicals mixture in their model was by Goodrich and colleagues, which used principal components to model third trimester BPA and phthalates in conjunction with targeted pyrosequencing of LINE-1 repetitive elements and *IGF2*, *H19* and Hydroxysteroid 11-Beta Dehydrogenase 2 (*HSD11B2)* among children aged 8–14 years [15]. In that study, it was found that children from mothers with higher third trimester urine BPA concentrations had increased DNA methylation of *IGF2* during peri-adolescence and that higher third trimester maternal urine monobenzylphthalate (mBzBP) and mono-isobutylphthalate (mIBP) concentrations were associated with increased DNA methylation of *H19* during peri-adolescence. In the primary components analysis, higher third trimester maternal urine mono-n-butylphthalate (mBP), mBzBP, mIBP and mono(3-carboxypropyl)-phthalate (mCPP) concentrations predicted increased *H19* methylation. We are unaware of any studies that have analyzed bisphenol or phthalate exposure from all three trimesters in relation to DNA methylation.

### Strengths and limitations

To our knowledge, we have conducted the first study exploring the associations of exposure to a mixture of phthalates and bisphenols at multiple time points during pregnancy with DNA methylation in cord blood. Using this novel mixture approach, we ensured that possible synergistic or antagonistic effects of the components of the mixture are taken into account.

We recognize that our ability to find associations was limited due to the relatively small study population. In addition, the Illumina Infinium Human Methylation450 BeadChip only covers 2% of all CpG sites in the DNA and we only assessed DNA methylation in cord blood, while other tissues could be more informative. The generalizability of our results could be limited due to the fact that the study population was relatively highly educated and only of European ancestry. However, even in this small population, we found potentially promising results that warrant further exploration of these associations in larger studies. As we assessed exposure during all three trimesters, we were able to explore possible vulnerable periods. Based on the number of associations, there is some indication that exposure during first and second trimester might be more relevant. It is known that early pregnancy in particular is a sensitive period for environmental exposures impacting DNA methylation [47]. However, due to the short biological half-lives of phthalates and bisphenols, it could be that our measurements do not accurately represent exposure during the whole trimester. These results should therefore be seen as exploratory and hypothesis-generating.

### Future perspectives

Our results support further exploration of exposure to a mixture of phthalates and bisphenols during pregnancy, preferably in a contemporary, larger, multi-ethnic cohort with multiple measures of exposure at different times during pregnancy.

## Conclusions

In this exploratory study, we found some suggestive associations for exposure to a mixture of non-persistent endocrine disruptors during first and second trimester of pregnancy with DNA methylation in cord blood. This study underscores the need for larger contemporary studies to further explore the association of mixtures of phthalates and bisphenols with DNA methylation.

## Supplementary Information

**Additional file 1. **Additional file containing supplemental figures and tables.** Fig. S1. **Flowchart of participants included in the study.** Fig. S2. **Manhattan plot of associations between a mixture of phthalates and bisphenols during first, second and third trimester with DNA methylation at birth.** Table S1. **Urine concentrations of phthalates and bisphenols during pregnancy in non-participants.** Table S2. **CpGs with p-values <1.0 * 10^–5^ from epigenome-wide association study of a mixture of phthalates and bisphenols in maternal urine during first, second and third trimester and DNA methylation in cord blood.** Table S3. **CpGs with p-values <1.0  * 10^–5^ from epigenome-wide association study of a mixture of phthalates and bisphenols in maternal urine during first, second and third trimester and DNA methylation in cord blood.** Table S4. **CpGs with p-values <1.0  * 10^–5^ from epigenome-wide association study of a mixture of phthalates and bisphenols in maternal urine during first, second and third trimester and DNA methylation in cord blood among boys.** Table S5. **CpGs with p-values <1.0  * 10^–5^ from epigenome-wide association study of a mixture of phthalates and bisphenols in maternal urine during first, second and third trimester and DNA methylation in cord blood among girls.** Table S6. **CpGs with p-values <1.0  * 10^–5^ from epigenome-wide association study of a mixture of phthalates and bisphenols in maternal urine averaged over pregnancy and DNA methylation in cord blood in the total group and among boys and girls specifically.

## Data Availability

The data that support the findings of this study are not publicly available due to privacy or ethical restrictions but are available from the corresponding author on reasonable request.

## Abbreviations

EDCs: Endocrine disrupting chemicals
EWAS: Epigenome-wide association study
FAM183A: Family with sequence similarity 183 member A
GHRHR: Growth hormone-releasing hormone receptor
HPCAL1: Hippocalcin-like 1
HSD11B2: Hydroxysteroid 11-beta dehydrogenase 2
IGF2: Insulin-like growth factor 2
IQR: Interquartile range
LOD: Limit of detection
mBP: Mono-*n*-butylphthalate
mBzBP: Monobenzylphthalate
mCPP: Mono(3-carboxypropyl)-phthalate
mIBP: Mono-isobutylphthalate
MICE: Multivariate imputation by chained equations
PEX10: Peroxisomal biogenesis factor 10
SD: Standard deviation
SE: Standard error
SNP: Single nucleotide polymorphism
SNRPB: Small nuclear ribonucleoprotein polypeptides B and B1
TRERF1: Transcriptional-regulating factor 1
