# Supplementary material for: Fetal exposure to phthalates and bisphenols and DNA methylation at birth: the Generation R Study
Source: Clin Epigenetics. 2022 Oct 10;14:125. doi: 10.1186/s13148-022-01345-0 (PMC9552446; doi:10.1186/s13148-022-01345-0)
Supplement: Supplementary file 1 — Additional file 1. Additional file containing supplemental figures and tables. Fig. S1. Flowchart of participants included in the study. Fig. S2. Manhattan plot of associations between a mixture of phthalates and bisphenols during first, second and third trimester with DNA methylation at birth. Table S1. Urine concentrations of phthalates and bisphenols during pregnancy in non-participants. Table S2. CpGs with p-values <1.0 * 10–5 from epigenome-wide association study of a mixture of phthalates and bisphenols in maternal urine during first, second and third trimester and DNA methylation in cord blood. Table S3. CpGs with p-values <1.0 * 10–5 from epigenome-wide association study of a mixture of phthalates and bisphenols in maternal urine during first, second and third trimester and DNA methylation in cord blood. Table S4. CpGs with p-values <1.0 * 10–5 from epigenome-wide association study of a mixture of phthalates and bisphenols in maternal urine during first, second and third trimester and DNA methylation in cord blood among boys. Table S5. CpGs with p-values <1.0 * 10–5 from epigenome-wide association study of a mixture of phthalates and bisphenols in maternal urine during first, second and third trimester and DNA methylation in cord blood among girls. Table S6. CpGs with p-values <1.0 * 10–5 from epigenome-wide association study of a mixture of phthalates and bisphenols in maternal urine averaged over pregnancy and DNA methylation in cord blood in the total group and among boys and girls specifically. [file 13148_2022_1345_MOESM1_ESM.docx]

**Supplemental materials**

**Fetal exposure to phthalates and bisphenols and DNA methylation at birth.**

**The Generation R Study**

Chalana M. Sol, MD, Abigail Gaylord, MPH, Susana Santos, PhD, Vincent W.V. Jaddoe, MD, PhD, Janine F. Felix, MD, PhD^$^, Leonardo Trasande, MD, MPP^$^

*^$^ These authors contributed equally.*

**Fig. S1.** Flowchart of participants included in the study.

**Fig. S2**. Manhattan plot of associations between a mixture of phthalates and bisphenols during first, second and third trimester with DNA methylation at birth.

**Table S1.** Urine concentrations of phthalates and bisphenols during pregnancy in non-participants.

**Table S2.** CpGs with p-values <1.0 * 10^-5^ from epigenome-wide association study of a mixture of phthalates and bisphenols in maternal urine during first, second and third trimester and DNA methylation in cord blood.

**Table S3**. CpGs with p-values <1.0 * 10^-5^ from epigenome-wide association study of a mixture of phthalates and bisphenols in maternal urine during first, second and third trimester and DNA methylation in cord blood.

**Table S4.** CpGs with p-values <1.0 * 10^-5^ from epigenome-wide association study of a mixture of phthalates and bisphenols in maternal urine during first, second and third trimester and DNA methylation in cord blood among boys.

**Table S5.** CpGs with p-values <1.0 * 10^-5^ from epigenome-wide association study of a mixture of phthalates and bisphenols in maternal urine during first, second and third trimester and DNA methylation in cord blood among girls.

**Table S6.** CpGs with p-values <1.0 * 10^-5^ from epigenome-wide association study of a mixture of phthalates and bisphenols in maternal urine averaged over pregnancy and DNA methylation in cord blood in the total group and among boys and girls specifically.

**Fig. S1.** Flowchart of participants included in the study.

| Mothers with information on exposure to phthalates and bisphenols during pregnancy with singleton live-born children  1405 | |  |  |
| --- | --- | --- | --- |
|  |  |  | Excluded: Mothers in whom information on exposure to phthalates and bisphenols is missing for at least one time point in pregnancy  n = 26  First trimester: n = 9  Second trimester: n = 14  Third trimester: n = 13 |
|  |  |  |  |
| Mothers with information on exposure to phthalates and bisphenols at all three time points in pregnancy with singleton live-born children  n = 1379 | |  |  |
|  |  |  | Excluded: Children without cord blood DNA methylation at birth  n = 1073 |
|  |  |  |  |
| Mothers with information on exposure to phthalates and bisphenols at all three time points in pregnancy and their singleton live-born children with DNA methylation measured in cord blood  n = 306 | |  |  |

**Fig. S2**. Manhattan plot of associations between a mixture of phthalates and bisphenols during first, second and third trimester with DNA methylation at birth.

**
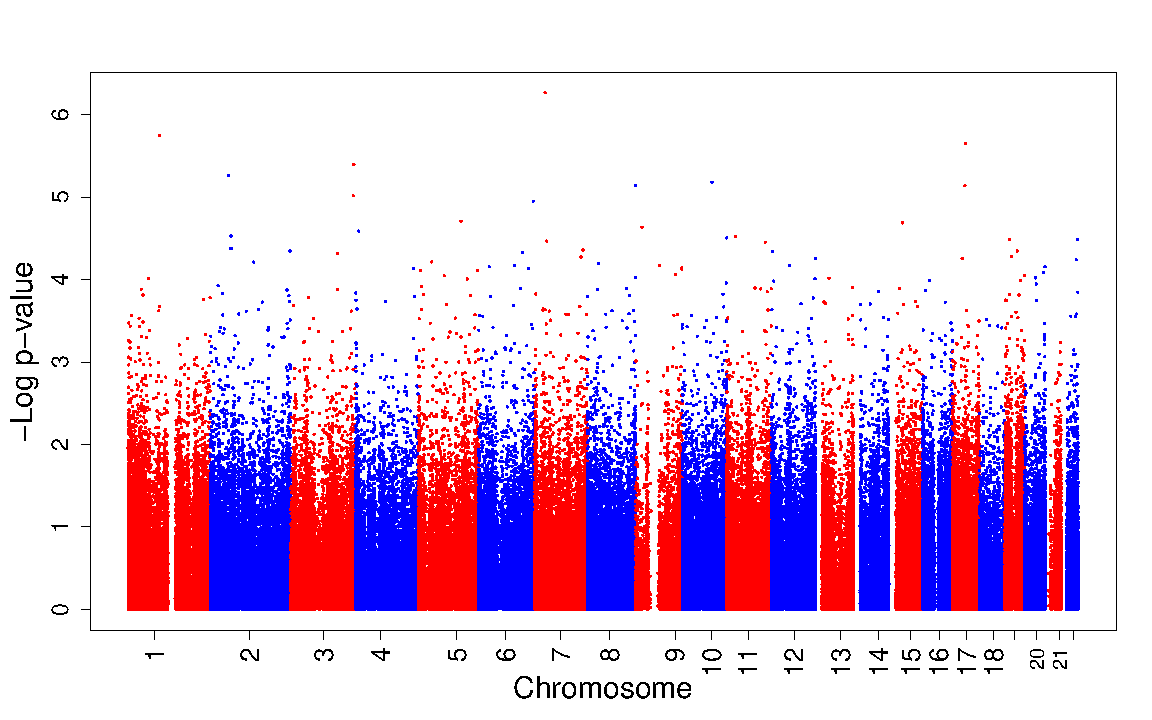
**
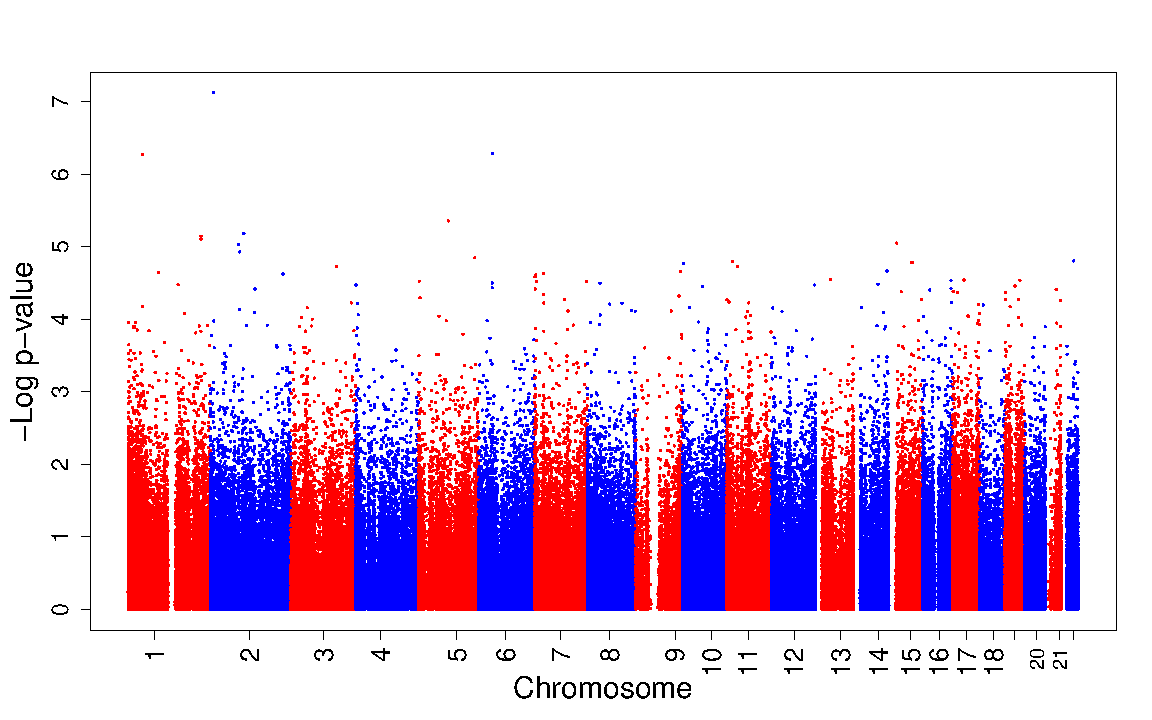

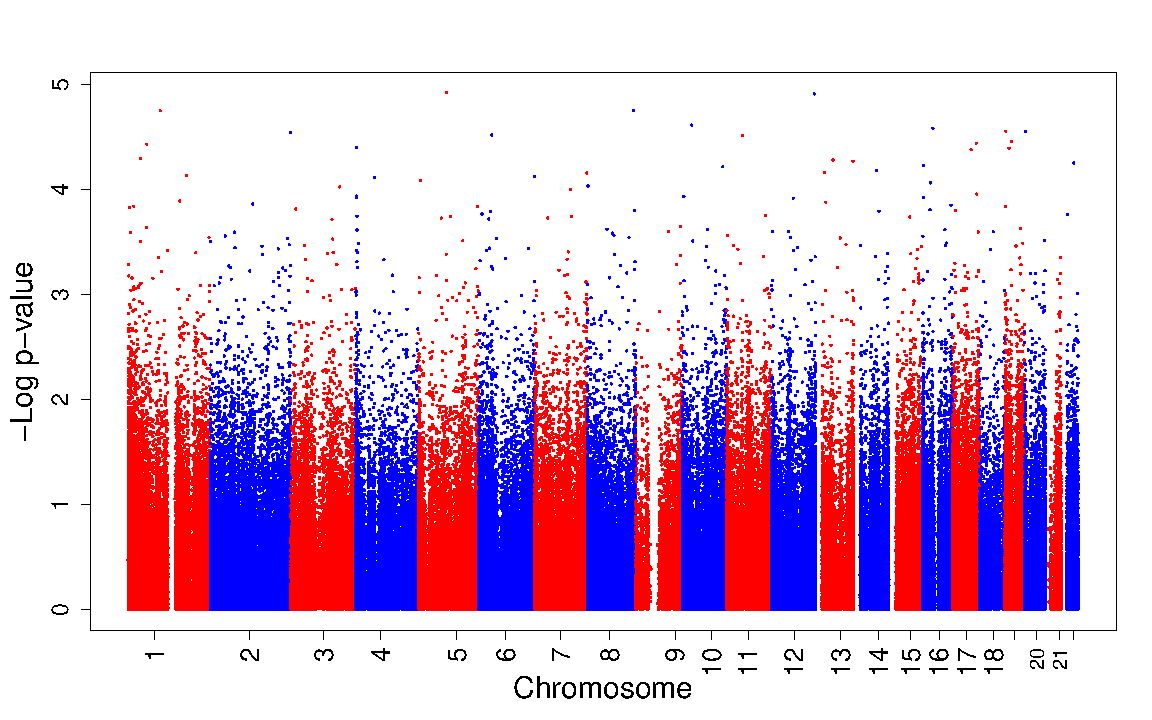


C

B

A

Manhattan plot of associations between a mixture of phthalates and bisphenols during first (A), second (B) and third (C) trimester with DNA methylation at birth for the model unadjusted for demographic covariates. In all Manhattan plots, the x-axis represents the autosomal chromosomes, the y-axis represents the –log_10_ of the p-value and the dots represent CpGs.

**Table S1.** Urine concentrations of phthalates and bisphenols during pregnancy in non-participants.

|  | **First trimester** | | | **Second trimester** | | | **Third trimester** | | |
| --- | --- | --- | --- | --- | --- | --- | --- | --- | --- |
|  | **Median (25^th^-75^th^ percentile)**  **Participants** | **Median (25^th^-75^th^ percentile)**  **Non-participants** | **Percentage <LOD**  **Non-participants** | **Median (25^th^-75^th^ percentile)**  **Participants** | **Median (25^th^ – 75^th^ percentile)**  **Non-participants** | **Percentage <LOD**  **Non-participants** | **Median (25^th^-75^th^ percentile)**  **Participants** | **Median (25^th^ – 75^th^ percentile)**  **Non-participants** | **Percentage <LOD**  **Non-participants** |
| Phthalic Acid (PA) (nmol/L) | 349.2 (195.4 – 844.3) | 336.7 (179.0 – 706.1) | 0.4 | 953.2 (381.6 – 1558.6) | 915.3 (371.4 – 1806.4) | 0.1 | 345.7 (187.1 – 715.6) | 448.4 (214.7 – 836.2)* | 0.4 |
| Monomethylphthalate (mMP) (nmol/L) | 28.3 (14.8 – 52.4) | 31.7 (15.5 – 55.6) | 0.1 | 18.5 (9.2 – 34.1) | 19.5 (10.6 – 35.2) | 0.1 | 16.8 (9.4 – 36.6) | 23.9 (11.7 – 45.9)* | 0.4 |
| Monoethylphthalate (mEP) (nmol/L) | 671.5 (198.0 – 2412.9) | 726.2 (218.3 – 2515.3) | 0.1 | 330.5 (123.2 – 1057.6) | 394.2 (130.1 – 1222.8) | 0 | 591.3 (207.8 – 1775.7) | 709.9 (238.4 – 2285.6) | 0 |
| Mono-isobutylphthalate (mIBP) (nmol/L) | 84.2 (38.3 – 157.8) | 100.9 (44.4 – 225.6)* | 0.2 | 35.6 (18.9 – 66.4) | 42.1 (21.7 – 89.6)* | 0 | 58.9 (33.6 – 115.0) | 90.2 (44.4 – 194.5)* | 0.3 |
| Mono-n-butylphthalate (mBP) (nmol/L) | 68.5 (30.8 – 124.5) | 73.5 (30.4 – 143.3) | 0.8 | 41.0 (24.4 – 75.1) | 45.8 (25.2 – 91.4) | 0 | 45.7 (23.9 – 78.8) | 57.3 (29.3 – 120.1)* | 0.2 |
| Monobenzylphthalate (mBzBP) (nmol/L) | 24.6 (8.8 – 43.6) | 22.1 (9.0 – 48.2) | 8.7 | 17.4 (7.2 – 33.6) | 22.3 (9.6 – 48.7)* | 1.2 | 9.9 (3.3 – 19.1) | 13.3 (4.9 – 27.7)* | 0.2 |
| Mono-(2-ethyl-5-carboxy-pentyl)phthalate (mECPP) (nmol/L) | 46.8 (26.3 – 92.9) | 55.0 (26.8 – 106.0) | 0.2 | 33.2 (18.7 – 59.2) | 35.5 (18.5 – 69.3) | 0.1 | 51.7 (26.8 – 90.3) | 60.8 (32.1 – 117.4)* | 0 |
| Mono-(2-ethyl-5-hydroxy-hexyl)phthalate (mEHHP) (nmol/L) | 35.1 (16.5 – 73.8) | 42.9 (20.5 – 80.7) | 0.2 | 19.1 (10.6 – 36.8) | 19.1 (10.0 – 37.8) | 0.1 | 33.0 (15.9 – 58.0) | 36.0 (18.2 – 73.1) | 0.2 |
| Mono-(2-ethyl-5oxohexyl)phthalate (mEOHP) (nmol/L) | 22.6 (10.4 – 45.7) | 27.4 (12.4 – 54.9)* | 0 | 26.5 (14.2 – 56.7) | 25.2 (12.2 – 56.9) | 0 | 22.5 (12.1 – 43.4) | 25.8 (13.5 – 49.4)* | 0.1 |
| Mono-[(2-carboxymethyl)-hexyl] phthalate (mCMHP) (nmol/L) | 42.4 (23.3 – 74.3) | 47.7 (25.0 – 90.2)* | 0.1 | 12.3 (7.1 – 23.4) | 13.7 (7.3 – 24.3) | 0.2 | 9.1 (5.0 – 17.4) | 12.0 (6.4 – 22.3)* | 1.2 |
| Mono(3-carboxypropyl)- phthalate (mCPP) (nmol/L) | 5.2 (3.0 – 10.4) | 5.9 (3.1 – 11.2) | 0 | 3.6 (2.0 – 6.6) | 3.5 (2.1 – 6.9) | 0 | 6.6 (3.7 – 12.1) | 7.5 (3.9 – 12.8) | 0.1 |
| Bisphenol A (BPA) (nmol/L) | 6.2 (1.6 – 14.8) | 4.6 (1.0 – 11.7)* | 21.9 | 5.2 (2.4 – 12.2) | 5.9 (2.7 – 13.6) | 6.5 | 6.0 (2.9 – 11.0) | 6.8 (2.6 – 14.1) | 10.3 |

Values represent medians (25^th^-75^th^ percentiles). Absolute urine concentration of the limit of detection (in nmol/L urine) and individual exposures (in nmol/L urine) with concentrations below the limit of detection imputed as limit of detection/square root of 2. Only values that have at least 75% above the limit of detection in all trimesters are presented in this table.

LOD, limit of detection.

* p-value < 0.05 when comparing participants and non-participants.

**Table S2.** CpGs with p-values <1.0 * 10^-5^ from epigenome-wide association study of a mixture of phthalates and bisphenols in maternal urine during first, second and third trimester and DNA methylation in cord blood.

| **First trimester** | **CpG** | **Chr** | **Position** | **Gene** | **Effect** | **SE** | **p-value** | **Flag^#^** |
| --- | --- | --- | --- | --- | --- | --- | --- | --- |
|  | cg05058973 | 7 | 31002599 | *GHRHR* | -1.20 * 10^-2^ | 2.37 * 10^-3^ | 7.08 * 10^-7^ | 0 |
|  | cg17091056 | 2 | 54315563 |  | -4.90 * 10^-3^ | 1.02 * 10^-3^ | 2.29 * 10^-6^ | 0 |
|  | cg19600538 | 1 | 95329706 | *SLC44A3* | -5.88* 10^-3^ | 1.27 * 10^-3^ | 5.79 * 10^-6^ | 0 |
|  | cg17196805 | 17 | 37796442 | *STARD3* | 6.58 * 10^-3^ | 1.45 * 10^-3^ | 7.86 * 10^-6^ | 1 |
| **Second trimester** | **CpG** | **Chr** | **Position** | **Gene** | **Effect** | **SE** | **p-value** | **Flag^#^** |
|  | cg00141688 | 2 | 10517352 | *HPCAL1* | 1.59 * 10^-2^ | 2.93 * 10^-3^ | 1.21 * 10^-7^ | 0 |
|  | cg15961211 | 1 | 43613440 | *FAM183A* | 3.65 * 10^-3^ | 7.11 * 10^-4^ | 5.32 * 10^-7^ | 0 |
|  | cg20840540 | 6 | 42363749 | *TRERF1* | -1.28 * 10^-2^ | 2.52 * 10^-3^ | 7.54 * 10^-7^ | 0 |
|  | cg18410444 | 5 | 89825535 | *LYSMD3* | -3.19 * 10^-3^ | 6.71 * 10^-4^ | 3.09 * 10^-6^ | 0 |
|  | cg06951677 | 1 | 221061837 |  | -2.31 * 10^-2^ | 5.09 * 10^-3^ | 8.08 * 10^-6^ | 0 |
|  | cg25464265 | 1 | 221063484 |  | -1.54 * 10^-2^ | 3.41 * 10^-3^ | 8.74 * 10^-6^ | 0 |
|  | cg20722537 | 2 | 86422644 | *IMMT* | 2.24 * 10^-3^ | 4.95 * 10^-4^ | 8.99 * 10^-6^ | 1 |
|  | cg27239243 | 2 | 101945867 |  | 1.51 * 10^-2^ | 3.35 * 10^-3^ | 9.00 * 10^-6^ | 0 |
|  | cg01961447 | 22 | 38221095 | *GALR3* | -1.85 * 10^-3^ | 4.09 * 10^-4^ | 9.11 * 10^-6^ | 0 |

There were no CpGs presented for third trimester, as none reached our uncorrected p-value cut-off of <1.0 * 10^-5^. There were no associations that reached significance (p-value <0.05) after further FDR-adjustment of the p-value for multiple testing including the three trimesters.

^#^ We have indicated probes that map to DNA containing a single nucleotide polymorphism (SNP), repetitive sequence elements or DNA harboring an insertion or deletion with a ‘1’ in this column.

**Table S3**. CpGs with p-values <1.0 * 10^-5^ from epigenome-wide association study of a mixture of phthalates and bisphenols in maternal urine during first, second and third trimester and DNA methylation in cord blood.

| **First trimester** | **CpG** | **Chr** | **Position** | **Gene** | **Effect** | **SE** | **p-value** | **Flag^#^** |
| --- | --- | --- | --- | --- | --- | --- | --- | --- |
|  | cg05058973 | 7 | 31002599 | *GHRHR* | -1.19 * 10^-2^ | 2.31 * 10^-3^ | 5.41 * 10^-7^ | 0 |
|  | cg19600538 | 1 | 95329706 | *SLC44A3* | -6.06 * 10^-3^ | 1.24 * 10^-3^ | 1.80 * 10^-6^ | 0 |
|  | cg22585042 | 17 | 39942456 | *JUP* | -4.92 * 10^-3^ | 1.02 * 10^-3^ | 2.26 * 10^-6^ | 0 |
|  | cg11201710 | 3 | 192232468 | *FGF12* | -1.17 * 10^-2^ | 2.50 * 10^-3^ | 4.02 * 10^-6^ | 0 |
|  | cg17091056 | 2 | 54315563 |  | -4.65 * 10^-3^ | 1.00 * 10^-3^ | 5.48 * 10^-6^ | 0 |
|  | cg20714462 | 10 | 90640409 | *STAMBPL1* | 1.61 * 10^-3^ | 3.50 * 10^-4^ | 6.58 * 10^-6^ | 0 |
|  | cg00065088 | 8 | 144943738 | *EPPK1* | 5.98 * 10^-3^ | 1.31 * 10^-3^ | 7.26 * 10^-6^ | 0 |
|  | cg17196805 | 17 | 37796442 | *STARD3* | 6.47 * 10^-3^ | 1.42 * 10^-3^ | 7.27 * 10^-6^ | 1 |
|  | cg21588838 | 3 | 190152481 | *TMEM207* | -8.50 * 10-3 | 1.89 * 10^-3^ | 9.65 * 10^-6^ | 0 |
| **Second trimester** | **CpG** | **Chr** | **Position** | **Gene** | **Effect** | **SE** | **p-value** | **Flag^#^** |
|  | cg00141688 | 2 | 10517352 | *HPCAL1* | 1.60 *10^-2^ | 2.91 * 10^-3^ | 7.51 * 10^-8^ | 0 |
|  | cg20840540 | 6 | 42363749 | *TRERF1* | -1.29 * 10^-2^ | 2.51 * 10^-3^ | 5.17 * 10^-7^ | 0 |
|  | cg15961211 | 1 | 43613440 | *FAM183A* | 3.62 * 10^-3^ | 7.06 * 10^-4^ | 5.35 * 10^-7^ | 0 |
|  | cg18410444 | 5 | 89825535 | *LYSMD3* | -3.13 * 10^-3^ | 6.68 * 10^-4^ | 4.41 * 10^-6^ | 0 |
|  | cg27239243 | 2 | 101945867 |  | 1.53 * 10^-2^ | 3.34 * 10^-3^ | 6.55 * 10^-6^ | 0 |
|  | cg25464265 | 1 | 221063484 |  | -1.55 * 10^-2^ | 3.38 * 10^-3^ | 7.18 * 10^-6^ | 0 |
|  | cg06951677 | 1 | 221061837 |  | -2.30 * 10^-2^ | 5.06 * 10^-3^ | 7.86 * 10^-6^ | 0 |
|  | cg12532169 | 15 | 23932370 | *NDN* | 1.33 * 10-2 | 2.94 * 10^-3^ | 8.91 * 10^-6^ | 0 |
|  | cg20722537 | 2 | 86422644 | *IMMT* | 2.22 * 10-3 | 4.91 * 10^-4^ | 9.25 * 10^-6^ | 1 |

There were no CpGs presented for third trimester, as none reached our p-value cut-off of <1.0 * 10^-5^. There were no associations that reached significance (p-value <0.05) after further FDR-adjustment of the p-value for multiple testing including the three trimesters. The presented associations were adjusted for child’s sex, cell percentages and technical differences based on technical batch.

^#^ We have indicated probes that map to DNA containing a single nucleotide polymorphism (SNP), repetitive sequence elements or DNA harboring an insertion or deletion with a ‘1’ in this column.

**Table S4.** CpGs with p-values <1.0 * 10^-5^ from epigenome-wide association study of a mixture of phthalates and bisphenols in maternal urine during first, second and third trimester and DNA methylation in cord blood among boys.

| **First trimester** | **CpG** | **Chr** | **Position** | **Gene** | **Effect** | **SE** | **p-value** | **Flag^#^** |
| --- | --- | --- | --- | --- | --- | --- | --- | --- |
|  | cg10148270 | 3 | 182400091 |  | 8.16 * 10^-3^ | 1.61 * 10^-3^ | 1.21 * 10^-6^ | 0 |
|  | cg08701028 | 2 | 54880931 | *SPTBN1* | -3.74 * 10^-3^ | 7.57 * 10^-4^ | 2.05 * 10^-6^ | 0 |
|  | cg06633429 | 5 | 76941396 |  | 6.96 * 10^-3^ | 1.44 * 10^-3^ | 3.61 * 10^-6^ | 0 |
|  | cg02157083 | 11 | 116662800 | *APOA5* | 1.29 * 10^-2^ | 2.69 * 10^-3^ | 3.80 * 10^-6^ | 0 |
|  | cg07678517 | 2 | 5944014 |  | 2.09 * 10^-2^ | 4.41 * 10^-3^ | 4.95 * 10^-6^ | 1 |
|  | cg16170767 | 6 | 99290737 |  | -1.96 * 10^-2^ | 4.16 * 10^-3^ | 5.46 * 10^-6^ | 1 |
|  | cg20400361 | 8 | 55014040 | *LYPLA1* | 6.85 * 10^-3^ | 1.47 * 10^-3^ | 7.00 * 10^-6^ | 0 |
| **Second trimester** | **CpG** | **Chr** | **Position** | **Gene** | **Effect** | **SE** | **p-value** | **Flag^#^** |
|  | cg03764767 | 1 | 2338210 | *PEX10* | -1.85 * 10^-2^ | 3.33 * 10^-3^ | 1.36 * 10^-7^ | 1 |
|  | cg25341937 | 6 | 17706893 | *NUP153* | 5.05 * 10^-3^ | 1.08 * 10^-3^ | 6.71 * 10^-6^ | 0 |
|  | cg16901788 | 1 | 247373896 |  | 6.45 * 10^-3^ | 1.40 * 10^-3^ | 8.42 * 10^-6^ | 0 |
|  | cg04737758 | 14 | 74824136 | *C14orf115* | -1.43 * 10^-2^ | 3.10 * 10^-3^ | 9.03 * 10^-6^ | 0 |
| **Third trimester** | **CpG** | **Chr** | **Position** | **Gene** | **Effect** | **SE** | **p-value** | **Flag^#^** |
|  | cg23462052 | 20 | 2452871 | *SNRPB* | 1.27 * 10^-2^ | 2.36 * 10^-3^ | 2.65 * 10^-7^ | 1 |
|  | cg04848682 | 14 | 77492060 | *C14orf4* | 2.19 * 10^-2^ | 4.73 * 10^-3^ | 7.99 * 10^-6^ | 0 |

There were no associations that reached significance (p-value <0.05) after further FDR-adjustment of the p-value for multiple testing including the three trimesters.

^#^ We have indicated probes that map to DNA containing a single nucleotide polymorphism (SNP), repetitive sequence elements or DNA harboring an insertion or deletion with a ‘1’ in this column.

**Table S5.** CpGs with p-values <1.0 * 10^-5^ from epigenome-wide association study of a mixture of phthalates and bisphenols in maternal urine during first, second and third trimester and DNA methylation in cord blood among girls.

| **First trimester** | **CpG** | **Chr** | **Position** | **Gene** | **Effect** | **SE** | **p-value** | **Flag^#^** |
| --- | --- | --- | --- | --- | --- | --- | --- | --- |
|  | ch.2.165639268R | 2 | 165931022 |  | -7.85 * 10^-3^ | 1.54 * 10^-3^ | 1.11 * 10^-6^ | 1 |
|  | cg18880500 | 1 | 53392492 | *SCP2* | 1.15 * 10^-2^ | 2.31 * 10^-3^ | 2.09 * 10^-6^ | 1 |
|  | cg04091961 | 19 | 622509 | *POLRMT* | 1.01 * 10^-2^ | 2.06 * 10^-3^ | 2.72 * 10^-6^ | 0 |
|  | cg23951780 | 4 | 184124500 | *WWC2* | 1.10 * 10^-2^ | 2.26 * 10^-3^ | 2.82 * 10^-6^ | 0 |
|  | cg10204707 | 19 | 53794711 | *BIRC8* | -5.61 * 10^-3^ | 1.16 * 10^-3^ | 3.52 * 10^-6^ | 0 |
|  | cg15504459 | 1 | 38478291 | *UTP11L* | -7.08 * 10^-3^ | 1.47 * 10^-3^ | 4.10 * 10^-6^ | 0 |
|  | cg17536465 | 5 | 126707043 | *MEGF10* | -8.60 * 10^-3^ | 1.79 * 10^-3^ | 4.16 * 10^-6^ | 1 |
|  | cg18781099 | 17 | 37911186 |  | -6.28 * 10^-3^ | 1.33 * 10^-3^ | 6.08 * 10^-6^ | 0 |
|  | cg05530751 | 10 | 45869477 | *ALOX5* | -1.00 * 10^-2^ | 2.15 * 10^-3^ | 7.23 * 10^-6^ | 0 |
|  | cg07137934 | 5 | 180230911 | *MGAT1* | -9.21 * 10^-3^ | 1.97 * 10^-3^ | 7.24 * 10^-6^ | 0 |
|  | cg26272651 | 8 | 1971644 |  | -2.79 * 10^-2^ | 5.98 * 10^-3^ | 7.28 * 10^-6^ | 1 |
|  | cg03413884 | 7 | 54610928 | *VSTM2A* | -2.71 * 10^-2^ | 5.82 * 10^-3^ | 7.72 * 10^-6^ | 0 |
| **Second trimester** | **CpG** | **Chr** | **Position** | **Gene** | **Effect** | **SE** | **p-value** | **Flag^#^** |
|  | cg15048660 | 3 | 192126935 | *FGF12* | 7.88 * 10^-3^ | 1.60 * 10^-3^ | 2.52 * 10^-6^ | 0 |
|  | cg17694848 | 6 | 112375200 | *WISP3* | -1.28 * 10^-2^ | 2.70 * 10^-3^ | 5.51 * 10^-6^ | 0 |
|  | cg17390129 | 6 | 90529507 | *MDN1* | 3.47 * 10^-3^ | 7.36 * 10^-4^ | 6.24 * 10^-6^ | 1 |
|  | cg13355574 | 10 | 134729942 |  | -9.09 * 10^-3^ | 1.93 * 10^-3^ | 6.46 * 10^-6^ | 0 |
|  | cg27129144 | 17 | 78848533 | *RPTOR* | 8.52 * 10^-3^ | 1.82 * 10^-3^ | 6.98 * 10^-6^ | 0 |
|  | cg17964921 | 5 | 872045 | *BRD9* | 1.26 * 10^-2^ | 2.71 * 10^-3^ | 8.25 * 10^-6^ | 0 |
| **Third trimester** | **CpG** | **Chr** | **Position** | **Gene** | **Effect** | **SE** | **p-value** | **Flag^#^** |
|  | cg07261186 | 10 | 81070689 | *ZMIZ1* | 2.67 * 10^-2^ | 5.68 * 10^-3^ | 6.53 * 10^-6^ | 0 |

There were no associations that reached significance (p-value <0.05) after further FDR-adjustment of the p-value for multiple testing including the three trimesters.

^#^ We have indicated probes that map to DNA containing a single nucleotide polymorphism (SNP), repetitive sequence elements or DNA harboring an insertion or deletion with a ‘1’ in this column.

**Table S6.** CpGs with p-values <1.0 * 10^-5^ from epigenome-wide association study of a mixture of phthalates and bisphenols in maternal urine averaged over pregnancy and DNA methylation in cord blood in the total group and among boys and girls specifically.

| **Total** | **CpG** | **Chr** | **Position** | **Gene** | **Effect** | **SE** | **p-value** | **Flag^#^** |
| --- | --- | --- | --- | --- | --- | --- | --- | --- |
|  | cg19287277 | 15 | 82337812 | *MEX3B* | -6.11 * 10^-3^ | 1.32 * 10^-3^ | 5.97 * 10^-6^ | 0 |
|  | cg06188496 | 1 | 9136476 | *SLC2A5* | 2.26 * 10^-2^ | 4.96 * 10^-3^ | 7.46 * 10^-6^ | 1 |
| **Boys** | **CpG** | **Chr** | **Position** | **Gene** | **Effect** | **SE** | **p-value** | **Flag^#^** |
|  | cg20400361 | 8 | 55014040 | *LYPLA1* | 9.50 * 10^-3^ | 1.80 * 10^-3^ | 4.93 * 10^-7^ | 0 |
|  | cg00025138 | 14 | 71275917 | *MAP3K9* | -2.57 * 10^-3^ | 5.01 * 10^-4^ | 8.64 * 10^-7^ | 0 |
|  | cg12117396 | 1 | 2438037 |  | -2.36 * 10^-2^ | 4.96 * 10^-3^ | 4.71 * 10^-6^ | 0 |
|  | cg10195011 | 12 | 48298798 | *VDR* | 7.33 * 10^-3^ | 1.57 * 10^-3^ | 6.99 * 10^-6^ | 0 |
|  | cg25087487 | 14 | 100626205 | *DEGS2* | -4.56 * 10^-3^ | 9.82 * 10^-4^ | 7.54 * 10^-6^ | 0 |
|  | cg00953403 | 17 | 74099816 | *EXOC7* | -1.15 * 10^-2^ | 2.47 * 10^-3^ | 7.63 * 10^-6^ | 1 |
|  | cg09162751 | 7 | 127033519 | *ZNF800* | 7.73 * 10^-3^ | 1.69 * 10^-3^ | 9.95 * 10^-6^ | 0 |
| **Girls** | **CpG** | **Chr** | **Position** | **Gene** | **Effect** | **SE** | **p-value** | **Flag^#^** |
|  | cg13344757 | 17 | 26904381 | *ALDOC* | 1.20 * 10^-2^ | 2.24 * 10^-3^ | 3.40 * 10^-7^ | 0 |
|  | cg04913443 | 14 | 21566084 | *ZNF219;C14orf176* | -7.00 * 10^-3^ | 1.33 * 10^-3^ | 5.13 * 10^-7^ | 0 |
|  | cg11723896 | 17 | 34136427 | *TAF15* | -1.05 * 10^-2^ | 2.02 * 10^-3^ | 6.95 * 10^-7^ | 1 |
|  | cg10074813 | 8 | 144637872 | *GSDMD* | 1.51 * 10^-2^ | 2.93 * 10^-3^ | 8.43 * 10^-7^ | 1 |
|  | cg15504459 | 1 | 38478291 | *UTP11L* | -8.02 * 10^-3^ | 1.55 * 10^-3^ | 8.60 * 10^-7^ | 0 |
|  | cg07080177 | 17 | 75243417 |  | -1.09 * 10^-2^ | 2.12 * 10^-3^ | 1.05 * 10^-6^ | 0 |
|  | cg02802590 | 2 | 96990549 | *ITPRIPL1* | -2.71 * 10^-2^ | 5.33 * 10^-3^ | 1.22 * 10^-6^ | 0 |
|  | cg21085625 | 2 | 120124643 | *C2orf76;DBI* | -6.28 * 10^-3^ | 1.28 * 10^-3^ | 2.64 * 10^-6^ | 0 |
|  | cg08328266 | 11 | 118992044 | *HINFP* | -6.55 * 10^-3^ | 1.33 * 10^-3^ | 2.65 * 10^-6^ | 0 |
|  | cg06162375 | 17 | 34839162 |  | -1.00 * 10^-2^ | 2.06 * 10^-3^ | 2.92 * 10^-6^ | 0 |
|  | cg27106233 | 10 | 74033853 | *DDIT4* | -6.23 * 10^-3^ | 1.28 * 10^-3^ | 3.00 * 10^-6^ | 0 |
|  | cg10095242 | 8 | 54163622 | *OPRK1* | -1.17 * 10^-2^ | 2.41 * 10^-3^ | 3.11 * 10^-6^ | 0 |
|  | cg04891921 | 3 | 134514173 | *EPHB1* | -9.16 * 10^-3^ | 1.88 * 10^-3^ | 3.15 * 10^-6^ | 0 |
|  | cg13860387 | 6 | 26456230 |  | 1.29 * 10^-2^ | 2.66 * 10^-3^ | 3.31 * 10^-6^ | 0 |
|  | cg16139011 | 11 | 9596224 | *WEE1* | -8.49 * 10^-3^ | 1.76 * 10^-3^ | 3.70 * 10^-6^ | 0 |
|  | cg03753642 | 8 | 75261222 | *GDAP1* | 1.26 * 10^-2^ | 2.60 * 10^-3^ | 3.79 * 10^-6^ | 1 |
|  | cg08581518 | 2 | 55747270 | *CCDC104* | -7.86 * 10^-3^ | 1.63 * 10^-3^ | 3.90 * 10^-6^ | 0 |
|  | cg00090309 | 7 | 156832367 |  | -2.26 * 10^-2^ | 4.68 * 10^-3^ | 3.94 * 10^-6^ | 1 |
|  | cg22585042 | 17 | 39942456 | *JUP* | -9.40 * 10^-3^ | 1.95 * 10^-3^ | 4.03 * 10^-6^ | 0 |
|  | cg10816846 | 12 | 132529397 | *EP400* | 8.94 * 10^-3^ | 1.86 * 10^-3^ | 4.26 * 10^-6^ | 1 |
|  | cg26780042 | 7 | 6515961 | *KDELR2* | 4.03 * 10^-2^ | 8.42 * 10^-3^ | 4.35 * 10^-6^ | 1 |
|  | cg04970434 | 17 | 4838096 | *GP1BA* | 1.63 * 10^-2^ | 3.40 * 10^-3^ | 4.37 * 10^-6^ | 0 |
|  | cg26483802 | 19 | 1830911 | *REXO1* | 1.02 * 10^-2^ | 2.13 * 10^-3^ | 4.93 * 10^-6^ | 0 |
|  | cg02866468 | 16 | 19842297 | *IQCK* | 1.60 * 10^-2^ | 3.38 * 10^-3^ | 5.35 * 10^-6^ | 1 |
|  | cg20309078 | 1 | 162531224 | *UAP1* | -9.26 * 10^-3^ | 1.95 * 10^-3^ | 5.50 * 10^-6^ | 0 |
|  | cg02889973 | 1 | 234977572 |  | 3.05 * 10^-2^ | 6.45 * 10^-3^ | 5.54 * 10^-6^ | 0 |
|  | cg21546610 | 17 | 3844820 | *ATP2A3* | 8.43 * 10^-3^ | 1.79 * 10^-3^ | 6.36 * 10^-6^ | 1 |
|  | cg04319606 | 2 | 26785290 | *C2orf70* | -9.93 * 10^-3^ | 2.11 * 10^-3^ | 6.52 * 10^-6^ | 0 |
|  | cg22190361 | 2 | 174886880 |  | 2.33 * 10^-2^ | 4.97 * 10^-3^ | 6.73 * 10^-6^ | 1 |
|  | cg13269166 | 7 | 142960561 | *GSTK1* | -1.05 * 10^-2^ | 2.25 * 10^-3^ | 8.05 * 10^-6^ | 0 |
|  | cg23426587 | 9 | 21335126 | *KLHL9* | -8.69 * 10^-3^ | 1.87 * 10^-3^ | 8.08 * 10^-6^ | 0 |
|  | cg27213509 | 2 | 176947228 | *EVX2* | -1.05 * 10^-2^ | 2.27 * 10^-3^ | 8.68 * 10^-6^ | 0 |
|  | cg16418817 | 21 | 40198485 |  | 2.95 * 10^-2^ | 6.41 * 10^-3^ | 9.22 * 10^-6^ | 0 |
|  | cg23018063 | 19 | 51531318 | *KLK11* | -1.35 * 10^-2^ | 2.94 * 10^-3^ | 9.64 * 10^-6^ | 1 |

^#^ We have indicated probes that map to DNA containing a single nucleotide polymorphism (SNP), repetitive sequence elements or DNA harboring an insertion or deletion with a ‘1’ in this column.
